# Good Effects of Ammoniacal Sulphate of Copper in Neuralgia of the Fifth Nerve (Tic Douloureux)

**Published:** 1879-11-20

**Authors:** 


### GOOD EFFECTS OF AMMONIACAL SULPHATE OF COPPER IN NEURALGIA OF THE FIFTH NERVE (TIC DOULOUREUX.)

Dr. Fereol having found several times obstinate cases of neuralgia of the fifth nerve, which had resisted a variety of other means, rapidly and completely cured by the administration of ammoniacal sulphate of copper, reports to the Academie de Medecine on the subject. The first case is that of a strong man, aged thirty-two, who had suffered so atrociously from terrible neuralgic crisis that on some days he was scarcely free for a few minutes at a time. Six teeth had been vainly extracted, and anti-neuralgic medication exhausted. He then tried ammoniacal sulphate of copper. The amelioration was considerable on the first day; on the second, the patient slept all night for the first time in two months; and at the end of ten days he left the hospital cured. A second case of supra-orbital neuralgia in a strong young man, occurring every morning and ceasing at noon, had been vainly treated by leeching, blistering and full doses of quinine. The ammoniacal sulphur of copper, given in a dose first of all of 0.10 and then 0.15 centigrammes daily, produced an immediate amelioration of pain, and the patient described himself as cured. The medication was continued for a week, and the neuralgia did not return. Similar effects were obtained by M. Fereol in a lady, aged forty-three, delicate, nervous, but not hysterical, suffering from persistent right hemicrania, with atrocious pain in the fifth pair of nerves, which drove her almost wild, and for which she had vainly tried quinine, aconitc, morphia, hypodermic injections, etc. Similar results were obtained in an old man, aged sixty, suffering for eighteen months from a horribly painful neuralgia, starting from the nasal branch of the fifth, and in whom local and general treatment by the oldest of anodynes and antiperiodics had been vainly tried. In this case the results were not permanent, the patient having an invincible dislike to the nausea produced by the sulphate of copper. The formula employed is the following: Distilled water, 100 grammes; syrup of orange flower or peppermint, 30 grammes; ammoniacal sulphate of copper, 0.10 to 0.15 centigrammes; to be taken in the course of twenty-four hours, especially during food, in order to avoid irritating the stomach. In one patient, the dose was raised to 60 centigrammes a day without any other inconvenience than slight gastric pain and a little diarrhœa. The medium dose was 0.10 to 0.15 centigrammes, which should be continued for from ten to fifteen days, even after the complete disappearance of the pains.—*London Medical Record*, 1879.

